# Raman Microspectroscopy Goes Viral: Infection Dynamics in the Cosmopolitan Microalga, *Emiliania huxleyi*

**DOI:** 10.3389/fmicb.2021.686287

**Published:** 2021-11-02

**Authors:** Elena Yakubovskaya, Tatiana Zaliznyak, Joaquín Martínez Martínez, Gordon T. Taylor

**Affiliations:** ^1^School of Marine and Atmospheric Sciences, Stony Brook University, Stony Brook, NY, United States; ^2^Bigelow Laboratory for Ocean Sciences, Boothbay, ME, United States

**Keywords:** Raman microspectroscopy, stable isotope probing, microalgae, virus, atomic force microscopy, infection, imaging

## Abstract

*Emiliania huxleyi* is a cosmopolitan member of the marine phytoplankton. This species’ capacities for carbon sequestration and sulfur mobilization make it a key player in oceanic biogeochemical cycles that influence climate on a planetary scale. Seasonal *E. huxleyi* blooms are abruptly terminated by viral epidemics caused by a clade of large DNA viruses collectively known as coccolithoviruses (EhVs). EhVs thereby mediate a significant part of material and energy fluxes associated with *E. huxleyi* population dynamics. In this study, we use spontaneous Raman microspectroscopy to perform label-free and non-invasive measurements of the macromolecular composition of individual virions and *E. huxleyi* host cells. Our novel autofluorescence suppression protocol enabled spectroscopic visualization of evolving macromolecular redistributions in individual *E. huxleyi* cells at different stages of EhV infection. Material transfer from *E. huxleyi* hosts to single EhV-163 virions was confirmed by combining stable isotope probing (SIP) experiments with Raman microspectroscopy. Inheritance of the host cells’ ^13^C-enriched isotopic signature was quantified based on red shifts of Raman peaks characteristic of phenylalanine’s phenyl ring. Two-dimensional Raman mapping of EhV-infected *E. huxleyi* cells revealed that the compact region producing an intense Raman DNA signal (i.e., the nucleus) in healthy *E. huxleyi* cells becomes diffuse during the first hours of infection. Raman DNA emissions integrated throughout individual cells decreased during the infection cycle. Our observations are consistent with EhV-163 degrading the host’s nuclear DNA, scavenging released nucleotides for its own genome replication, and shedding newly-produced virions prior to host lysis *via* budding.

## Introduction

The microalga *Emiliania huxleyi* is a biogeochemically important coccolithophorid (Prymnesiophyceae) that is a major participant in exporting carbon and calcium to the deep-sea and seabed (Balch, 2018). *Emiliania huxleyi* produces vast seasonal blooms with peak densities sometimes exceeding 10^7^ cells L^−1^ (van der Wal et al., 1995; Tyrrell and Merico, 2004) and sometimes spanning more than 100,000km^2^ of ocean surface waters (Brown and Yoder, 1994; Balch, 2018). These blooms affect the radiative balance of the sea surface at mesoscale dimensions (Holligan et al., 1983), play a key role in climate regulation by massive production of dimethylsulfoniopropionate, which is degraded to volatile dimethyl sulfide that triggers marine cloud formation (Shaw, 1983; Charlson et al., 1987), and contribute up to 70% of oceanic calcite production (Westbroek et al., 1993). All told, this makes *E. huxleyi* one of very few species whose population dynamics affect biogeochemistry and climate at nearly global scales.

More than one quarter of the biogenic carbon flux in the ocean is estimated to pass through the viral shunt (Wilhelm and Suttle, 1999). Several coccolithoviruses, collectively known as *E. huxleyi* viruses or EhV (Vardi et al., 2012; Laber et al., 2018), directly affect oceanic material fluxes by inducing massive lysis of *E. huxleyi* cells. EhV is arguably one of the few known viral groups whose propagation affects energy and material transfer on a planetary scale.

Coccolithoviruses fall within a monophyletic group of nucleocytoplasmic large DNA viruses (NCLDV) in the Phycodnaviridae family (Bratbak et al., 1993). They possess an icosahedral nucleocapsid (170–220nm diameter) and an external lipid envelope acquired by budding through the host’s cytoplasmic membrane (Martínez et al., 2007; Mackinder et al., 2009). EhVs’ genomes are large (376–421kb), and they carry unique sets of auxiliary metabolic genes (AMGs; Wilson et al., 2005), which can completely reprogram functioning of the host cell’s central carbon metabolism (Moniruzzaman et al., 2020).

Our understanding of EhVs’ interactions with their hosts is notably limited by the fact that the overwhelming majority of studies are based on observations of bulk samples of algal cell and virion populations. Population averaging may alias or mask subtle yet important variations among members of a genetically heterogeneous asynchronous population. Meanwhile, we are just beginning to appreciate how important phenotypic heterogeneity is in enabling microbial populations to persist in fluctuating environments, to react to external challenges and opportunities, and to proliferate in spatially structured microenvironments (Ackermann, 2015). Study of host-virus interactions at the single-cell level may considerably advance our knowledge about the effects of phenotypic heterogeneity.

Select methods used for single-cell study of algal host and virus interactions have proven to be instrumental in unravelling fine details of complex host-virus dynamics by disentangling cells experiencing different infection states, such as single-cell expression profiling (Ku et al., 2020). These methods are necessarily destructive, and therefore providing no information about spatial distribution of specific components in a cell. Various microscopic techniques, such as electron or atomic force microscopy, are capable of visualizing fine structural details, but provide very limited information on chemical composition of infected cells. Super-resolution fluorescence microscopy (Berjón-Otero et al., 2020) provides fine details of spatial distributions of targeted molecules in cells (Nathan and Daniel, 2019), yielding information unobtainable by other methods. However, specific fluorochrome tracers are required, which can significantly limit the method’s application. Recently, nano-SIMS was applied to measure isotopic compositions of individual *E. huxleyi* host cells and their EhV-207 viral particles and was complemented with BONCAT-based visualization of released viruses (Pasulka et al., 2018). Despite limitations of the individual methods summarized by the authors, combining them offer a novel and powerful approach to measuring viral particle production at the single particle scale. Methods that provide both structural and chemical information, that do not require label introduction, and can be applied to individual cells and viruses would be ideal for studying host-virus interactions. These requirements essentially are satisfied by confocal Raman microspectroscopy (Parab and Tomar, 2012).

Raman microspectroscopy measures inelastically-scattered photons from specimens illuminated by monochromatic light, i.e., a laser. The amount of energy transferred from laser photons to target molecules, and hence the spectral shift of Raman scattered photon emissions, is characteristic of the covalently-bound elements excited, bonding properties, and vibrational mode. Measurement of these laser-induced transitions in biological samples provides valuable information about common functional groups, such as phenylalanine’s aromatic ring or DNA’s phosphodiester bond. This enables measuring distribution of a cell’s most common macromolecules in their native state (Salzer et al., 2000; Lambert et al., 2006). Furthermore, molecular vibrations are very sensitive to the masses of the atoms involved in these oscillations. Consequently, any isotopic enrichment (e.g., ^13^C and ^15^N) of a Raman-active functional group can be detected as a pronounced red shift of its characteristic Raman-scattered photons. Therefore, when combined with various stable isotope probing (SIP) techniques, Raman microspectroscopy can be used to explore material transfer processes between the environment and cells and material movement within and between cells (Li et al., 2012, 2013; Taylor et al., 2017a).

Autofluorescence from chromophores, which are especially abundant in photosynthetic cells, has historically hampered application of Raman microspectroscopy to single-cell imaging. A number of methods for circumventing autofluorescence have proven effective in limited applications (reviewed by Wei et al., 2015). However, each solution has its own intrinsic limitations, e.g., (i) fluorescence quenching by prolonged laser photobleaching impedes data throughput, (ii) fluorescence-free surface-enhanced Raman scattering (SERS) requires infusing cells with Au or Ag nanoparticles (Le Ru et al., 2007; Wei et al., 2015), (iii) fluorescence-free resonance Raman scattering is limited to detecting the chromophores themselves (Taylor et al., 2017a; Taylor, 2019), and (iv) time-resolved avoidance of fluorescence requires sophisticated ultrafast pulsed lasers and gated detection (Ariese et al., 2009; Taylor, 2019). Recently, we developed a novel autofluorescence suppression method that combines chemical and photo-bleaching of entire samples prior to Raman interrogation that enables direct and efficient chemical mapping of algal cells using a conventional Raman microspectrophotometer and standard data processing protocols (Yakubovskaya et al., 2019).

Virions have been detected recently by technologically-demanding Raman approaches, such as tip-enhanced and coherent anti-Stokes Raman scattering, TERS and CARS, respectively (Cialla et al., 2008; Deckert et al., 2020). However, in this report, we present the first-ever spontaneous Raman spectra from single virions and spectral verification that virions inherit the isotopic signature of their ^13^C-labeled host cells. We also examine *E. huxleyi* host cells at different stages of EhV-163 intracellular propagation by Raman microspectroscopy. Two-dimensional (2-D) Raman chemical mapping of DNA and protein distributions in individual *E. huxleyi* cells indicate that EhV-163’s replication strategy may include degradation of host’s nuclear DNA and pirating the released nucleotides to build new viral genomes. Temporal changes in the 2-D Raman maps also suggest that virions are continuously shed *via* virion budding before the lytic event.

## Materials and Methods

More detailed descriptions of methods are presented in the Supplementary Material.

### Microalgal Cultivation, Virus Propagation, and Purification

*Emiliania huxleyi* (a non-calcifying strain CCMP374) was grown at 20°C in f/2-Si seawater medium and infected with the EhV-163 virus during exponential growth phase (~2×10^6^ cells per ml) with a virus-to-cell ratio (multiplicity of infection=MOI) of 0.2. Lysate was subjected to tangential flow filtration followed by isolation in an iodixanol (OptiPrep™) density gradient, followed by dialysis and additional chromatographic purification on a Capto™ Core 700 column.

### Atomic Force Microscopy and Transmission Electron Microscopy

Atomic Force Microscopic (AFM) imaging of EhV-163 samples was performed on an Innova® AFM (Bruker™, United States) in tapping mode with silicon tips. For electron microscopy purified viral samples were stained with uranyl acetate, and the grids were imaged on a Tecnai™ F20 transmission electron microscope operated at 80kV with a Gatan™ ORIUS® SC1000B CCD camera.

### Preparation of Viral Aggregates and Individual Virions for Raman Measurement

EhV-163 samples were fixed with 2% formaldehyde for 15min, filtered onto 0.02μm Whatman® Anopore™ Al_2_O_3_ membranes, rinsed with deionized water, and freeze transferred to a polished stainless steel microscope slide.

### Stable Isotope Probing

*Emiliania huxleyi* cultures (strain CCMP374) were grown in f/2-Si seawater medium supplemented with 20mM ^13^C-bicarbonate. Cells in mid-exponential growth phase were preserved (2% formaldehyde) and gently captured on polycarbonate membranes, stored at 4°C until processing. Isotopic enrichment of cells was determined by observing the red shift of the characteristic Phe Raman peak from 1,002 to 966cm^−1^ (corresponding to fully ^13^C-substituted phenyl ring), along with less intense peaks at 988 and 977cm^−1^ corresponding to partially ^13^C-substituted phenyl rings. Fractional abundances of ^13^C (*f*) in Phe pools were determined by curve deconvolution and peak fitting as described in Supplementary Material.

### Chemiphotobleaching of *E. huxleyi* Specimens

Filter-captured cells were treated with 3% hydrogen peroxide/visible light irradiation as described in (Yakubovskaya et al., 2019). After chemiphotobleaching, cells were freeze-transferred to mirror-finished stainless steel microscope slides and subjected to Raman microspectroscopic analysis (Taylor et al., 2017b).

### Raman Measurements, Spectral Analysis, and Imaging

Raman measurements were performed using a Renishaw® inVia™ confocal Raman microspectrophotometer and were processed using the WiRE™ 5.1 program package and Python scripts written in our lab (included in Supplementary Material). Two-dimensional mapping was performed after 2h of chemiphotobleaching and up to additional 3min laser bleaching of individual cells under a low magnification lens. Raman chemical maps were acquired with 4mW laser excitation (633nm) at the sample, in StreamHR mode at 0.5μm *x*-*y* step size, and a detector exposure time of 50s. For label-free detection of individual virions, five individual virions and 20 EhV-163 aggregates were interrogated, and spectra were presented with or without averaging. Macromolecular composition of viral-like particles was determined from spectra of five individual particles in each case, followed by averaging. For carbon flow measurements, 20 individual spectra of host cells and virions were collected. Detailed protocols for data collection, spectral data processing, and 2-D mapping protocols are presented in Supplementary Material. Raw spectral data are available upon request to the corresponding author.

## Results

### Novel EhV-163 Purification Protocol Produces High Quality Viral Samples

Virion sample quality appears to be critical to reliable Raman measurements. Therefore, we optimized a virion purification protocol, which produced high quality EhV-163 particles, largely free of cellular debris, and contaminants. The protocol (see Supplementary Figure S1) builds upon traditional steps for virus purification, such as filtration, concentration, and density gradient separation (OptiPrep™, iodixanol), followed by dialysis. OptiPrep™ was selected, because unlike CsCl or sucrose, it is nearly isosmotic with the virions, thereby minimizing possibility of damaging the viral envelope by osmotic shock. A Capto™ Core 700 column was used to further purify EhV-163 suspensions and remove iodixanol. Capto™ Core 700 multimodal chromatography resin enables size exclusion chromatographic separation of large biomolecular assemblies, like viruses, and concurrent scavenging of low molecular weight ionic mixtures in the sorbent’s interior.

Atomic Force Microscopic images of EhV-163 prepared using the above protocol show that the overwhelming majority of virion particles in their native state have a nearly spherical shape, their size distribution is unimodal, and have an average particle diameter of 161nm (Supplementary Figure S2). The smaller and larger size bins represent fragments and aggregated individuals, respectively. The diameter of uranyl stained transmission electron microscopic (TEM) images of EhV-163 is somewhat greater (190nm; Supplementary Figure S2C). The apparent discrepancy in virion size may result from flattening of particles on EM grids during uranyl staining. Both AFM and TEM images demonstrate that our protocol produces high quality viral suspensions that will likely be useful for future studies of large enveloped viruses.

### Spontaneous Raman Microspectroscopy Enables Direct Label-Free Detection of Individual Virions

To explore whether spontaneous Raman microspectroscopy was capable of detecting individual virions and of assessing the quality of our purified EhV-163 preparations, Raman spectra of viral aggregates were obtained first. Initial efforts to obtain Raman spectra from EhV-163 suspensions that were deposited and dried directly on polished stainless steel microscope slides were unsuccessful. Even though flow cytometry, AFM, and TEM confirmed high concentrations of viral particles, Raman signals were not forthcoming from these samples. We speculate that Raman-inactive NaCl in the Capto™ Core 700 eluent precipitated over virion particles as samples dried, thereby shielding them from laser interrogation. Washing EhV-163 virions captured on 0.02μm pore size membranes with deionized water followed by their freeze-transfer to stainless steel microscope slides resolved the problem. Strong Raman scattering was then readily detected from viral aggregates.

**Figure 1:**
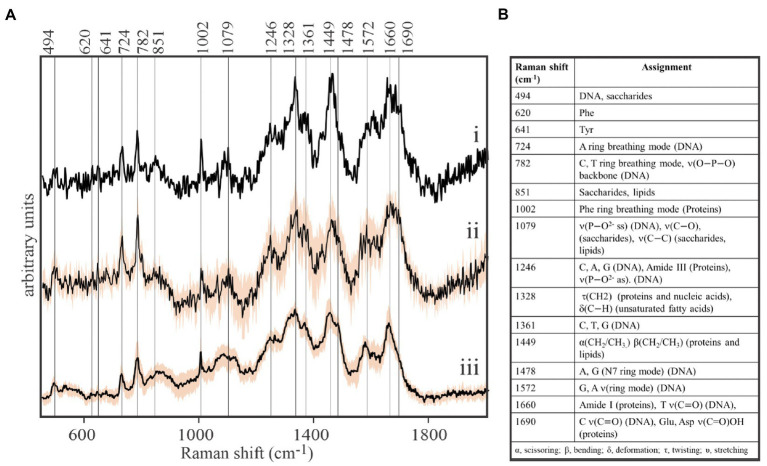
Direct, label-free detection of individual virions by spontaneous Raman microspectroscopy. **(A)** Stacked Raman spectra of purified formaldehyde-fixed coccolithovirus (EhV)-163 virions deposited on stainless steel microscope slides. All spectra clearly reveal the presence of peaks characteristic of the three main components of EhV-163: proteins, DNA, and lipids. (i) Spectrum of a single virion. (ii) Average spectrum from five individual virions. (iii) Average spectrum from 20 EhV-163 aggregates. Shading over spectra (ii) and (iii) represents one standard deviation (± 1SD) about the mean observed at every wavenumber for spectra from five individual virions or 20 viral aggregates. **(B)** Raman shift assignments reported for specific bonds and molecules (Movasaghi et al., 2007).

Our protocol was then pressed to its sensitivity limits to acquire spontaneous Raman spectra from spatially-isolated, individual viral-sized particles on the stainless steel substrates. While EhV-163 virion size is below the resolution limit of bright-field optical microscopy (0.2μm), they are nonetheless visible when spatially-isolated on a clean reflective surface. High quality Raman spectra were acquired from individual viral-sized particles that revealed the presence of characteristic peaks of the three main components of EhV-163: proteins, DNA, and lipids (Figure 1). In fact, signal-to-noise ratios and information content of the spectra are improved only marginally by averaging spectra from multiple individual virions or interrogating larger viral aggregates (Figure 1). All spectra have a sharp peak at 1,002cm^−1^ emanating from the phenylalanine (Phe) phenyl ring breathing mode, which is a Raman signature of proteins. Peaks characteristic of nucleic acids were apparent at 724cm^−1^ reportedly arising from the adenine (A) ring breathing mode in DNA/RNA and at 782cm^−1^ arising from phosphodiester backbone stretching mode and pyrimidine nucleobase (C and T/U) ring breathing mode (Movasaghi et al., 2007). A peak at 1,449cm^−1^ is indicative of the scissoring and/or bending mode of aliphatic methylene groups, a typical functionality in all lipids, and to a smaller extent in proteins (Movasaghi et al., 2007). Therefore, we conclude that spontaneous Raman microspectroscopy of a single or few virions provide reliable information about their essential components. Thus, it can be used for non-invasive and label-free studies of their composition, which enables study of compositional inhomogeneity of virion samples. To the best of our knowledge, these are the first-ever spontaneous Raman microspectroscopic measurements of individual viral particles that do not require less accessible instrumentation or signal amplification techniques, such as TERS (Cialla et al., 2008, Deckert et al., 2020).

### Analysis of Macromolecular Composition of Virions by Raman Spectroscopy

**Figure 2:**
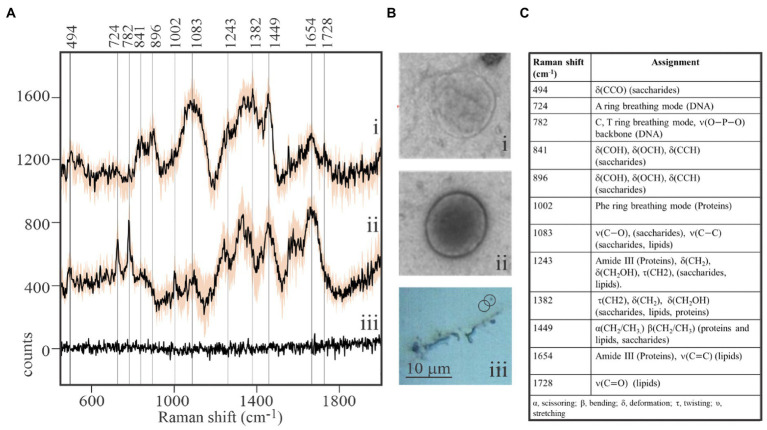
Macromolecular composition of viral-like particles. **(A)** Stacked spectra from purified, formaldehyde-fixed EhV-163 viral preparations illustrating differences between empty structures (i) and complete virions (ii). Spectra are means from five viral-sized targets and shaded area represents one standard deviation (±1SD) about the mean observed at each wavenumber. The absence of diagnostic peaks for DNA (724 and 782cm^−1^) and Phe (1,002cm^−1^) and presence of lipid peaks (1,382 and 1,449cm^−1^) in (i) suggest targets were empty envelopes or extracellular vesicles. Spectra from the stainless steel slide surface were obtained from areas devoid of virions and illustrate negligible background contributed by the substratum (iii). **(B)** Examples of transmission electron microscopic (TEM) images of an individual empty structure (i) and individual complete EhV-163 virion (ii) prepared from replicate samples. (iii) Reflected bright-field micrograph of areas on slide with virion aggregates and individual virions. **(C)** Raman shift assignments reported for specific bonds and molecules (Movasaghi et al., 2007).

While viral-sized particles can be detected at 1,000x magnification under reflected bright-field illumination, ascertaining the identity of such small particles by optical microscopy is impossible. We observed that Raman spectra obtained from individual putative viral particles sometimes yielded unexpected results (Figure 2). It was not uncommon to retrieve spectra from targets resembling Figure 2A (i) that showed a clear signature of aliphatic lipids at 1,382–1,449cm^−1^, but lacked characteristic DNA or protein peaks. The spectroscopic properties of complete virions appeared to be dramatically different (Figure 2A (ii)). Peaks characteristic of DNA in its common B form (724 and 782cm^−1^; Deng et al., 1999) were prominent in spectra of complete virions. As expected, DNA peaks were not found in spectra of particles that are interpreted to be empty viral envelopes or possibly extracellular vesicles. Instead, signatures for lipids, sugars, and proteins (841 and 896cm^−1^ – deformation of sugar’s COH; 1,382cm^−1^ – twisting and deformation of lipid and saccharide methylenes; and 1,449cm^−1^ – scissoring and bending of methylenes in lipids and proteins) dominated spectra of the empty membranous particles. Differences in Raman spectra of these two particle types are entirely consistent with differences in the physical appearance of images from replicate samples subjected to uranyl-stained TEM [Figure 2B (i) and (ii)]. Empty membranous particles and complete virions have nearly the same overall size and shape, but the former are markedly electron transmissive (transparent). In contrast, a dark core with consistent structure, most likely the uranyl-stained nucleocapsid, is clearly seen in complete virions. It is noteworthy that composite spectra obtained by averaging spectra of as few as five structurally complete virions or five empty particles appeared sufficient to resolve differences in the main diagnostic peaks, thereby demonstrating the very high sensitivity of our approach. Furthermore, spectra obtained from areas devoid of virions illustrate that the stainless steel slide sample substrate contributes negligible background Raman scattering to target spectra.

### Detecting Material Transfer Between *E. huxleyi* Host Cells and EhV-163 Viruses by SIP-Raman

Stable isotope probing is a well-known technique used to trace material transfer processes in biological systems (Taylor et al., 2017a). We used Raman microspectroscopy to document EhV-163 virions’ inheritance of their ^13^C-labeled host’s isotopic signature.

Historically, isotopic enrichments in photoautotrophic algal cells have been measured by resonance Raman microspectroscopy, which measures the scattered emissions from isotopologues of highly absorbing chromophores, such as carotenoids (Li et al., 2012; Taylor et al., 2017a). The laser energy used is close to electronic transition bands of these pigments, which produces significant Raman signal amplification and overwhelms incidental fluorescence from other chromophores. This approach, however, is not applicable to the present study, because virions are devoid of natural pigments suitable for resonance Raman measurements. Spontaneous Raman microspectroscopy, however, is equally applicable to algal cells and free virions, capable of measuring red shifts in omnipresent vibrational modes, such as those emanating from aromatic rings of amino acid side chains, nucleobases, or lipids.

**Figure 3:**
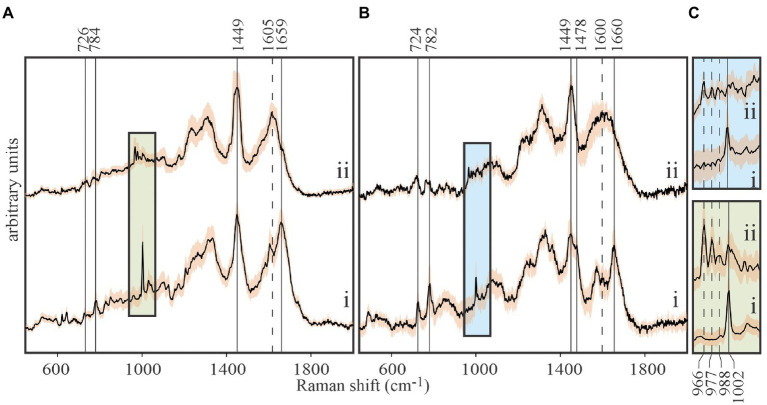
Tracing carbon flow from ^13^C-labeled *Emiliania huxleyi* to EhV-163 viruses by stable isotope probing (SIP) and Raman microspectroscopy. **(A)** Stacked spectra of *E. huxleyi* grown in unlabeled media (i), and grown in media with ^13^C-bicarbonate (20mM) as the sole inorganic carbon source (ii). **(B)** Stacked spectra of purified EhV-163 virions obtained from unlabeled (i) and ^13^C-labeled (ii) *E. huxleyi* hosts. Solid vertical lines show the Raman peaks that undergo significant reds shift due to ^13^C enrichment. **(C)** Magnified spectral region containing Phe’s ring breathing mode peaks for the host (lower panel) and EhV-163 virions (upper panel). Emission peaks from the four observed isotopologues are specified by vertical lines. Shading over spectra represents ±1SD about the mean observed at every wavenumber for spectra from 20 cells or 20 viral aggregates.

Although spontaneous Raman emissions are intrinsically weak, this technique has been successfully applied to measurement of isotopic enrichments in highly fluorescent bacterial cells that required prolonged photobleaching to suppress autofluorescence before spectral acquisition (Huang et al., 2007). While this approach was successful for single point measurements of bacterial cells, its application to surveying multiple points in larger cells, such as *E. huxleyi*, is not practical. Our recently developed chemiphotobleaching approach has proven effective in irreversibly suppressing *E. huxleyi* autofluorescence for entire samples that include multiple cells. Once quenched, high quality spontaneous Raman spectra were obtained that enabled measuring isotopic enrichments of *E. huxleyi* cells (Figure 3).

Isotopic enrichment of *E. huxleyi* was achieved by growing cultures in f/2 media enriched with 20mM ^13^C-bicarbonate as the only source of carbon. Isotopic enrichment could be monitored reliably by the Phe peak in Raman spectra at 1,002cm^−1^ for natural isotopic abundances, due to it rarely being obscured by emissions from other functional groups, and its narrow bandwidth and high intensity (Figure 3A). Observed stepwise red shifts due to partial ^13^C incorporation in Phe’s phenyl ring (1,002→988→977→966cm^−1^) perfectly reproduced theoretical predictions from expected isotopologue distributions. Isotopically-enriched *E. huxleyi* cells were infected with EhV-163 virus, and 5days after infection released EhV-163 virions were separated, purified, and analyzed by Raman microspectroscopy.

**Figure 4:**
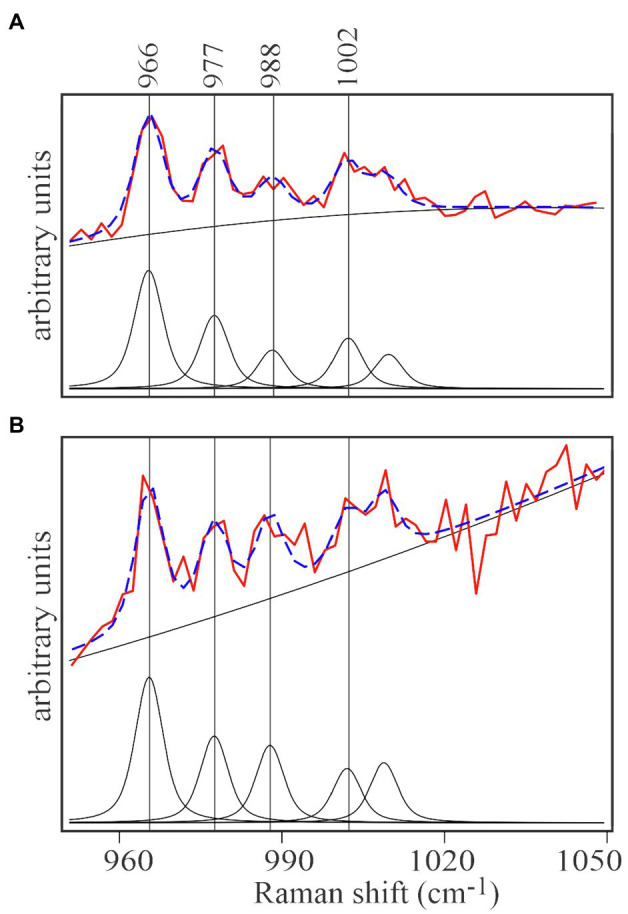
Curve-fitting analysis of isotopic signatures of ^13^C enriched *E.huxleyi* cells **(A)** and the EhV-163 released from them **(B)**. Both panels represent the averaged spectral region including the four characteristic Phe isotopologue peaks of ^13^C-enriched *E.huxleyi* and EhV-163 presented in Figure 3C. After baseline correction and normalization, contribution of each isotopologue was determined by curve deconvolution and peak fitting (lower spectrum in each panel). Fitting results of the isotopologue distribution are illustrated by the broken blue lines superimposed on the actual observations (red spectrum in each panel).

Spectra of unlabeled *E. huxleyi* and cells grown on ^13^C-enriched bicarbonate are shown in Figure 3A. Comparing those with spectra from EhV-163 virions released from their host cells (Figure 3B) demonstrates that isotopic signatures of both virus samples were nearly identical to those of their respective hosts. Fidelity of isotopic signature inheritance serves as a direct demonstration of material transfer between the host and its virus. Figure 4 illustrates how Raman scattering from Phe isotopologues in free EhV-163 virions nearly duplicates that observed in their ^13^C-labeled *E. huxleyi* hosts. In accordance with earlier reports (Li et al., 2013), four isotopologues are clearly seen, which correspond to the unlabeled phenyl ring (the band at 1,002cm^−1^), a two ^13^C substitution (989cm^−1^), a four ^13^C substitution (978cm^−1^), and a fully labelled phenyl R group (966cm^−1^). Curve-fitting of these isotopologue peaks enables calculation of ^13^C fractional abundance (*f*) in Phe, which presumably represents the entire protein pool as well as other macromolecules as populations equilibrate with their medium. Such calculations yield an *f_host_* of 0.65±0.07 for *E. huxleyi* and an *f_virus_* of 0.63±0.01 for the EhV-163 released (Figure 4). Essentially isotopic fractional abundances of virions and host are indistinguishable to within analytical uncertainty.

### *In vivo* Infection Detection: Visualization of *E. huxleyi*-EhV-163 Virocell Development

Cursory examination of 2-D Raman maps of *E. huxleyi* cells before and after EhV-163 infection in the ^13^C-SIP experiment indicated that intracellular distributions of macromolecules were distinct among these populations. This observation suggested that Raman mapping might provide label-free evidence of viral activity directly within individual intact host cells. Efficient high resolution mapping of microalgae requires that all autofluorescence be suppressed prior to data acquisition. We found that our chemiphotobleaching pre-treatment (dilute H_2_O_2_/white light irradiation; Yakubovskaya et al., 2019) irreversibly suppressed autofluorescence in an entire sample without causing any demonstrable damage or redistribution of intracellular components in the specimen.

Semi-quantitative comparisons of molecular distributions in 2-D chemical maps also require a robust normalization protocol. An internal standard for normalization should be a spectral peak from a molecule whose abundance in cells is not prone to large variations at different cell cycle stages or at different infection stages. The best candidate is the Phe peak at 1,002cm^−1^: Phe is an abundant amino acid, present in almost all proteins, and as stated above is easily resolved and quantified by Raman microspectroscopy.

**Figure 5:**
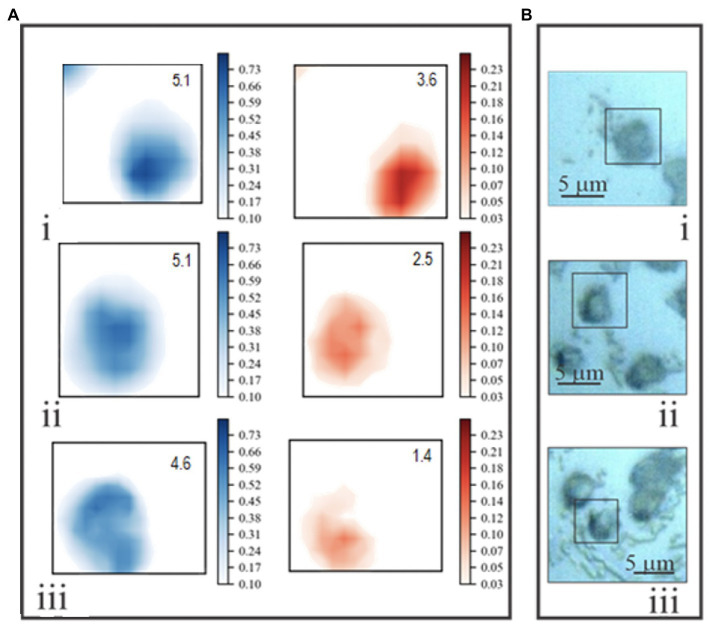
**(A)** Examples of two-dimensional Raman chemical maps of uninfected *E. huxleyi* cells (i), and cells 24 (ii) and 48 (iii) hours after being infected with EhV-163. Intensities at 1,002cm^−1^ (protein – blue) and 782cm^−1^ (DNA – red) were mapped using a standard shared color intensity scale for all cells. Integrated pixel intensities (arbitrary units) of “protein portraits” and “DNA portraits” are shown in the top right corner of each box. **(B)** Reflected bright-field optical micrographs of the same specimens. Rectangles frame areas of each specimen subjected to Raman mapping.

Normalized chemical maps of uninfected *E. huxleyi* cells and EhV-163-infected cells illustrate spectral manifestations of different degrees of viral propagation within *E. huxleyi* cells. Maps of cells were created based on 2-D distributions of 782 and 1,002cm^−1^ peak intensities. The Phe 1,002cm^−1^ peak was used to map protein distributions. The 782cm^−1^ peak was used to indicate nucleic acids, but its origin is less clear-cut, because it may originate from stretching vibration of phosphodiester inter-nucleotide bonds in B form DNA or thymine/cytosine ring beathing vibrations (Thomas et al., 1986). Therefore, this peak could indicate presence of both DNA (thymine, cytosine, and phosphodiester peaks) and RNA (cytosine only). Attribution of the 782cm^−1^ peak to DNA is, however, supported by the facts that the predominant RNA conformation state is the A form, and its phosphodiester bonds have an intense characteristic peak at 813cm^−1^ (Erfurth et al., 1975). However, no Raman peak is evident at that wavenumber in any of our *E. huxleyi* spectra. Furthermore, EhV-163 virions lack RNA while their spectral profile in the 700–800cm^−1^ region closely resembles that of their hosts. Accordingly, we conclude that RNA’s contribution to emissions at 782cm^−1^ is minor and that this vibrational mode primarily emanates from B form DNA’s phosphodiester backbone and pyrimidines. Hence, we refer to the 782 and 1,002cm^−1^ chemical maps as “DNA portraits” and “protein portraits” of the cell, respectively. Comparison of these images with the reflected bright-field micrographs demonstrates that the “protein portrait” effectively represents the overall shape of the cell (Figure 5). Furthermore, protein distributions and intensities are reasonably uniform in cells during all stages of infection development. In contrast, DNA distributions change significantly after infection by EhV-163. Qualitatively, the changes are consistent with the classical scenario of intracellular viral propagation, which includes viral-induced rupture of nuclear membrane at early stages of infection. “DNA portraits” of healthy *E. huxleyi* cells show that most DNA content is concentrated in a relatively compact and dense core, presumably in the nucleus (Figure 5i). However, 1day after infection, the DNA-rich region becomes diffuse (Figure 5ii). Two days after infection, the dense DNA-rich core completely disappears (Figure 5iii). Given that our unifected *E. huxleyi* cells were not synchronously dividing and all cells did not become infected simultaneously, cell-to-cell variability was evident among a population of “DNA portraits” at any given time point (Supplementary Figure S3). However, quantitative analysis of integrated pixel intensities in “DNA portraits” demonstrate that total DNA abundance in *E. huxleyi* cells decreased by more than 2-fold during viral propagation (*p*<0.01; ANOVA), while mean protein abundance remained more or less constant (*p*>0.60; ANOVA; Supplementary Figure S4). At 72h post-infection, cellular protein and DNA abundances were most variable among cells, which we attribute to some cells lysing and decomposing.

## Discussion

Recent progress in biological applications of Raman spectroscopy has been driven especially by signal enhancement techniques, such as surface-enhanced and tip-enhanced Raman scattering approaches, SERS and TERS, respectively. SERS and TERS increase Raman scattering efficiencies over normally weak spontaneous Raman scattering (~1 scattered photon per 10^6^–10^7^ excitation photons) by orders of magnitude. For example, TERS was recently combined with CARS to distinguish between individual H1N1 and coxsackievirus B3 RNA viruses (Deckert et al., 2020). Tellingly, an RNA signal was detected in the small coxsackievirus B3 virions (20–30nm diameter), but not in the much larger H1N1 virion (150–400nm diameter) enveloped in a lipid bilayer (Deckert et al., 2020). This illustrates that TERS primarily interrogates surface moieties because the TERS effect requires that target molecules are proximal to the gold AFM tip to experience the enhanced electromagnetic field (Le Ru et al., 2007). While providing high sensitivity and extraordinary spatial resolution (~20nm) for surface chemistry characterization, TERS does not appear to be well-suited for interrogating internal moieties of particles larger than about 100nm. Furthermore, TERS and CARS require rare instrumentation not generally available in many biological laboratories, e.g., an optically coupled AFM, a second femtosecond “pump” laser, and a time-gated detector (Deckert et al., 2020). Therefore, conventional Raman microspectroscopy remains the practical method of choice for non-invasive study of viral-host interactions.

In this study, we characterized key aspects of virus–host interactions between EhV-163 and *E. huxleyi* at the single-cell level. We demonstrated that spontaneous Raman microspectroscopy can measure macromolecular composition of individual virions, and its sensitivity is sufficient to discriminate reliably between ostensibly complete virions and empty envelopes. This capability enables more systematic future studies characterizing the relative abundances of fully functional, incompletely assembled, and empty viral-like particles. Such studies can shed light on important and currently unknown aspects of EhV’s reproductive cycle. Furthermore, the same technique can be applied to explore the mysteries of why *E. huxleyi* releases extracellular vesicles, which are currently believed to play an important, yet undescribed role in intercellular communication (Schatz et al., 2017).

It is worth noting that while we used a commonly studied non-calcified *E. huxleyi* strain (e.g., Bidle et al., 2007; Evans et al., 2007; Johns et al., 2019; Gilg et al., 2016; Pasulka et al., 2018; Goode et al., 2019; Knowles et al., 2020), we expect the results from this study are also representative of infection of calcified strains. The infection dynamics and virion production are not significantly impacted by whether the host cells are calcified or not (Paasche, 2001; Haunost et al., 2020). However, variations in strains of hosts and virions and host physiology more likely account for reported differences in infection success and dynamics (Allen et al., 2007; Kegel et al., 2013). Lastly, most hosts of large viruses are non-calcified (Fischer, 2006). Therefore, we conclude that the non-calcified *E. huxleyi* strain is a better subject for application of Raman microspectroscopy to visualize macromolecular redistributions in individual intact host cells at different stages of infection.

By applying SIP-Raman microspectroscopy, we verified material transfer between isotopically enriched algal host and released virions. As expected, virions inherited the isotopic signature of their hosts with high fidelity, because all newly synthesized viral components are derived from resources produced by the host’s biosynthetic machinery. The distribution of isotopologues (double, quadruple, and fully ^13^С substituted phenyl rings) in Phe was almost identical in both host and released virions. As suggested previously (Li et al., 2013), the labeling pattern is consistent with the biochemical pathway of Phe synthesis starting from ^13^C-enriched glucose, which is a key intermediate of photosynthetic conversion of inorganic bicarbonate into organic matter. The excellent correspondence observed between algal and viral ^13^C isotopic signatures demonstrates that Raman microspectroscopy can be used for quantitative measurement of material transfer between unicellular algae and their viruses. We underscore that isotopic signature inheritance was apparent from spontaneous Raman emissions from biologically ubiquitous functional groups, such as the phenyl ring of Phe and DNA signatures. Therefore, our measurements are not dependent on chromophores, such as carotenoids or other pigments that are not universally present among all biota. Using universally distributed reporter molecules enables measurement of material transfer between a whole range of trophic partners and antagonists, including transfer between prokaryotic and eukaryotic cells, symbiotic partners, and from host cells to viruses. In particular, this approach may be applied to measure release rates of new viral particles and virion sourcing experiments, as well as for quantitative studies of material fluxes through the viral shunt at the community level.

Intracellular infection of individual intact microalgae was also detectable by Raman microspectroscopy. We observed significant variations in spatial distribution of Raman emission at 782cm^−1^ in *E. huxleyi* cells at different stages of viral propagation. The 2-D Raman maps in Figure 5A (red) describe distributions of cellular material that we interpret to contain B-form DNA, primarily attributable to host and viral ds-DNA. Raman maps of uninfected *E. huxleyi* cells exhibited a compact region of intense emissions at 782cm^−1^, whose size and position correspond to the cell’s nucleus. One day post infection, Raman emissions at 782cm^−1^ became blurred, and nuclear content appeared to be redistributed throughout the cell, which most likely resulted from EhV-163 virion rupturing the nucleus.

After EhV-163 disrupts *E. huxleyi’s* nuclear membranes, the following alternative replication scenarios are plausible. They differ in their main source of nucleotides for EhV genome replication, and in timing of the virions’ exit from the host.

1. Viral invader redirects the host’s transcription pathways to transcribe its own genome and divert all available resources to synthesis of new virions. That includes activation of a *de novo* nucleotide synthesis pathway to increase the dNTPs pool level, needed for synthesis of multiple sizable viral genomes. New virions are released from host cell at late stage of infection. In this scenario, a surge in Raman emissions at 782cm^−1^ would be expected early after infection, because of active *de novo* synthesis of new pyrimidine bases.
2. Same as scenario (1), but new virions are shed from the host cell *via* budding. In this case, virions shed from the host carry newly synthesized DNA away, so intensity of 782cm^−1^ Raman emission of the host cell would be expected to be somewhat elevated during viral replication until virion shedding commences when intracellular DNA pools would decline.
3. EhV-163 activates a DNA hydrolysis pathway that leads to quick cleavage of host DNA. Concurrently, a nucleotide salvage pathway is activated, which converts the products of DNA hyrdolysis into dNTPs, followed by their incorporation into viral genomes. In this scenario, the 782cm^−1^ Raman emission intensity would remain relatively invariant, because few or no new nucleotides would be produced. A relatively constant nucleotide inventory would remain in the host’s cytoplasm in the form of newly synthesized virions. New virions would then be released during a lytic event.
4. Same as scenario (3), but new virions are continuously shed from the host cell during replication *via* budding, so a steady decrease of the overall Raman emission at 782cm^−1^ would be observed.

The observed variations in the overall intensity and spatial distribution of Raman emissions at 782cm^−1^ are most consistent with scenario #4. This conclusion is in agreement with published observations. For example, some nucleocytoplasmic viruses are reported to carry endonucleases that cleave the host DNA at very early stages of infection, before viral replication commences (Agarkova et al., 2006). The cleavage may serve as a tool for partial or full suppression of transcription of the host’s genome. Such suppression was observed in *E. huxleyi* cells infected by the EhV-201 virus, occurring at early stages of infection (8–9h post infection; Ku et al., 2020). EhV-201 and EhV-163 are phylogenetically related and share genomic homologies (Allen et al., 2006; Nissimov et al., 2012). Therefore, it is quite likely that EhV-163 suppresses the host’s transcription, much like EhV-201, early in infection process. This is also consistent with observations of Chloroviruses, large DNA viruses in the same family as EhV, that use endonucleases to degrade host DNA early in infection (Agarkova et al., 2006). Furthermore, activation of autophagy processes was reported to play an essential role in propagation of the EhV-201 virus (Schatz et al., 2014). We propose that disruption of transcription is achieved by extensive cleavage of the host’s nuclear DNA. It is plausible that nucleotide salvage is an essential pathway activated by EhV-163 to convert host DNA into dNTPs for viral DNA replication.

Activation of a nucleotide salvage pathway is especially important for large viruses, such as EhVs. Their large genome (~400kb, Allen et al., 2006), suggests that availability of nucleotides may be a bottleneck to their replication process. EhV’s replication strategy may rely on the host’s genome as an essential source of costly nucleotides. The size of *E. huxleyi* genome is estimated to be ~142Mbp for haploid cells (Read et al., 2013), and, accordingly, ~282Mbp for diploid cells, which means each *E. huxleyi* nucleus contains at least ~560 million deoxynucleotides. Therefore, *E. huxleyi’s* genome alone could provide a sufficient number of nucleotides to produce ~700 complete virions. qPCR measurement of EhV-201 propagation demonstrated that 100 extracellular and 250 intracellular virions are produced per *E. huxleyi* cell 70h after infection, or a total of 350 just prior to the lytic phase (Schatz et al., 2014). In the case of EhV-163 that has a mean estimated burst size of 630 viral particles per cell (range=400–1,000), the host’s nuclear DNA is more than adequate to provide the required dNTP inventory to support observed burst sizes (Castberg et al., 2002).

Destruction and repurposing of genomic DNA in infected *E. huxleyi* cells appear to be the most energetically favorable strategy, because it achieves two goals simultaneously. Firstly, the host’s metabolic pathways are redirected toward viral replication without conflicting host transcripts. Secondly, nucleotides required for EhV-163 replication are recycled from the host’s DNA rather than synthesized *de novo*, which is a lengthy and energy-demanding pathway.

Finally, the fact that the EhV clade has very large genomes, in fact bigger than that of some bacteria, is consistent with the proposed replication scenario. Clearly, small viruses are totally dependent on the cellular machinery encoded by the host’s genome. Genomes of those viruses have just a few essential genes that complement host’s protein pool, so their replication may require transcription of some essential host’s genes. In contrast, the genomes of EhVs contain hundreds of genes, including essential genes for DNA and RNA metabolism (Ku et al., 2020). The most plausible reason for carrying so many genes is to eliminate the viruses’ dependence on a functional host genome when the infected cell is converted into a reaction vessel devoid of the host’s nuclear genome control. If our hypothesis is correct, viral replication relies exclusively on viral genes directing utilization of cellular resources available at the infection’s start, while energy and reducing power continue to be produced by functional chloroplasts and mitochondria.

Our study demonstrates that by using conventional Raman microspectroscopic equipment and relatively simple experiments, it is possible to obtain valuable information about the composition of submicron-sized particles, including individual virions of large nucleocytoplasmic DNA viruses, their envelopes, and possibly extracellular vesicles or similar structures. Using spontaneous Raman microspectroscopy, it is possible to directly observe variations in macromolecular distributions in infected cells, which provides solid support for possible scenarios for viral infection progression, and potentially, can be used in more extensive future studies of various aspects of interactions between viruses and their eukaryotic hosts. We believe this study provides a convincing demonstration of the many capabilities of Raman microspectroscopic techniques, and we anticipate it will be recognized as a valuable complement to other approaches, such as single-cell transcriptomics.

## Data Availability Statement

The original contributions presented in the study are included in the article/Supplementary Material; further inquiries can be directed to the corresponding author.

## Author Contributions

EY, TZ, JM, and GT designed the experiments. EY and TZ performed most of the experiments and analyzed the data. GT supervised the project. EY and GT wrote the manuscript. All authors contributed to the article and approved the submitted version.

## Funding

This research was supported by the Gordon and Betty Moore Foundation Marine Microbiology Initiative, through grant GBMF-5604 awarded to GT and JM.

## Conflict of Interest

The authors declare that the research was conducted in the absence of any commercial or financial relationships that could be construed as a potential conflict of interest.

## Publisher’s Note

All claims expressed in this article are solely those of the authors and do not necessarily represent those of their affiliated organizations, or those of the publisher, the editors and the reviewers. Any product that may be evaluated in this article, or claim that may be made by its manufacturer, is not guaranteed or endorsed by the publisher.
