# Supplementary material for: Raman Microspectroscopy Goes Viral: Infection Dynamics in the Cosmopolitan Microalga, Emiliania huxleyi
Source: Front Microbiol. 2021 Nov 2;12:686287. doi: 10.3389/fmicb.2021.686287 (PMC8593419; doi:10.3389/fmicb.2021.686287)
Supplement: Supplementary file 1 [file Data_Sheet_1.PDF]

Supplementary Material

Raman Microspectroscopy Goes Viral: Infection Dynamics in the Cosmopolitan Microalga, *Emiliania huxleyi*

Elena Yakubovskaya[1], Tatiana Zaliznyak[1], Joaquín Martínez Martínez[2],

and Gordon T. Taylor[1]*

[1]School Marine and Atmospheric Sciences, Stony Brook University, Stony Brook, NY 11794 USA

[2] Bigelow Laboratory for Ocean Sciences, East Boothbay, Maine 04544, USA.

*** Correspondence:**
Corresponding author: *email: gordon.taylor @stonybrook.edu*

1 Supplementary Data

Materials and Methods

Microalgal cultivation. Cultures of the prymnesiophyte, *Emiliania huxleyi* (CCMP374 - non-calcifying strain) were grown in batch mode at 20°C in f/2-Si medium prepared in filtered (0.22 µm) seawater (Guillard and Ryther, 1962). Cultures were incubated in Fisher suspension cell culture flasks (250 ml) with vent caps on a rotating platform that assured uniform light exposure (48–63 µmol quanta m^{-2} s^{-1}) during 12:12 h light/dark cycles. Cells samples were preserved in 2% borate-buffered formaldehyde (final concentration) and stored at 4°C.

Viral infection and virion purification. When *E. huxleyi* culture achieved cell densities of $1{\times}10^6$ cells mL^{-1}, a 500ml culture was infected with the EhV-163 virus, using a 1:100 volumetric ratio of viral lysate to culture. Subsamples of infected cells were taken every 12 hours for 7 days, preserved in 2% borate-buffered formaldehyde (final conc.) and stored at 4°C. After the infected host culture cleared (6 days), the lysate was filtered through a 0.8 µm nitrocellulose filter (to remove cell debris), followed by passage through a 0.45 µm PES filter. Lysate was concentrated 50x by tangential flow filtration with a Vivaflow 50 cartridge (50 KDa MWCO). The concentrate yielded ~7 x 10^9 EhV ml^{-1}. An OptiPrep™ (STEMCELL Technologies ™) density gradient and ultracentrifugation were used for further purification of the virus. A range of concentrations of OptiPrep™ solutions were prepared

using physiological buffer ("PB" = 150mM NaCl, 1mM EDTA, 20 mM HEPES pH 8.0) as the diluent. To form a 6-step gradient the following technique was used: solutions of OptiPrep™ dilutions were introduced at the bottom of open-topped ultracentrifuge tubes beginning with the least dense solution first. The gradient consisted of: 1 ml of 20%, 2 ml of 25%, 2 ml of 30%, 2 ml of 35%, 2 ml of 40%, 1 ml of 50% (vol/vol) OptiPrep™ solutions. Gradient was blended by leaving for 2 h at room temperature to create a continuous gradient. Concentrated EhV samples (2 ml of sample per 15 ml OptiPrep™ gradient per tube) were loaded on top of the continuous OptiPrep™ gradient and ultracentrifuged using SW-41 rotor on a Beckman Optima XL-90 Ultracentrifuge for 4h at 100,000x g and a slow deceleration program below 4000 rpm. Virus bands are easily visible with a simple flashlight. For harvesting a single band in the density gradient we punctured the side or bottom with a needle. To separate the EhV-163 from OptiPrep™ matrix, sample was dialyzed (Float-A-Lyzer™ G2 100kD cut off) three times against 1L of PB. EhV-163 suspensions were further purified on a Capto™ Core 700 chromatographic column that has both size exclusion and affinity binding properties. Three ml of Capto™ Core 700 resin was equilibrated with 10ml of PB and EhV-163 was purified by batch method to remove traces of iodixanol. Purified virus samples were stored as aliquots at 4°C in the dark. Our workflow is summarized in Fig. SI1A.

Virus quantification and quality analysis. Quality of the purified viral sample was determined by measuring the size and shape of individual viral particles deposited on a pristine mica surface using AFM (Fig. SI2). Analysis of multiple AFM fields revealed that the size frequency distribution of EhV-163 was more or less unimodal, with an average diameter of ~161 nm, corresponding to fully functional virions (Fig SI1B). Variation in particle size was moderate, and can be attributed to interactions with the mica surface, non-uniform drying, some fragmentation, and some aggregation. Transmission electron microscopy (TEM) images of EhV-163 reveal more fine details of its internal structure (Fig. SI2C). A magnified view illustrates a nearly spherical external shape of this enveloped virion, as well as the intactness of its lipid envelope. Taken together, these observations demonstrate that our preparation and purification procedures are non-destructive, and provide good quality samples that are sufficiently clean for experimental purposes (For comparison to published AFM viral study (Bocklitz *et al*, 2014).

Preparation of viral aggregates and individual virions for Raman measurement. EhV-163 samples were fixed with 2% formaldehyde for 15 min, filtered onto 0.02 μm Whatman® Anopore™ Al_2O_3 membranes, rinsed with deionized water and freeze-transferred to a polished stainless steel microscope slide. (Yakubovskaya *et al*, 2019)

Atomic Force Microscopy (AFM). Once purified as described above, 10 μl subsamples of EhV-163 suspensions were deposited onto a freshly cleaved mica substrate. Dried adsorbed samples were washed three times with 50 μl of deionized-distilled water, blotted and then air-dried. Dried samples were imaged using Innova® AFM (Bruker™, USA). Bruker™ FMV-A silicon probes were used in tapping mode with a cantilever resonance frequency about 320 kHz and cantilever spring pressure constant of 42 N m^{-1}.

Negative staining Transmission Electron Microscopy. Aliquots (3 µl) of purified EhV-163 suspensions were deposited on 300-mesh carbon-coated copper grids (Ted Pella™) that were previously glow discharged for 10 s. After 1 min, excess viral suspension was blotted away with filter paper and stained with a 1% (w/v) uranyl acetate solution. Grids were imaged on a Tecnai™ F20 transmission electron microscope operated at 80kV with a Gatan™ ORIUS® SC1000B CCD camera. Images were acquired at a magnification of 23,000x, 49,000x and 80,000x.

Stable isotope probing (SIP). *E. huxleyi* cultures were grown in f/2-Si seawater media in which 2 mM dissolved inorganic carbon was augmented with 10 mM of ^{13}C-bicarbonate. The ^{12}C- and ^{13}C-bicarbonate solutions (Cambridge Isotope Laboratories, Inc. Andover, MA; 99% ^{13}C, 97% chemical purity) were prepared as 1M working stocks. Nutrient and bicarbonate solutions were aseptically added directly to autoclaved 0.22 µm filtered seawater. Cultures were grown in 125 ml sterile non–vented PC Erlenmeyer flasks with same total DIC concentrations in ^{12}C and ^{13}C treatments with 10mM ^{12}C-bicarbonate solutions added to control flasks and 10mM ^{13}C-bicarbonate solutions added to experimental flasks. Cultures were grown for 10 days and subsampled daily to determine growth rates by Aquafluor™ fluorimeter. Subsamples (3 ml) were fixed with 2% borate-buffered formaldehyde (final concentration) and stored at 4°C for Raman spectroscopic analysis. (Taylor *et al*, 2017)

Chemiphotobleaching protocol. Appropriate volumes of the fixed *E. huxleyi* cultures (1-3 ml) were passed through 0.2 µm Millipore™ GTTP polycarbonate membranes. Membranes were air-dried for 10 min on Whatman™ 3MM CHR filter paper and placed in 1 inch Petri dishes with 3 % hydrogen peroxide (diluted from 30% in MilliQ H_2O) under a bright white (3695K), 251 lumens LED desk lamp (Hampton Bay® #1000 052 866) for 2 h. To ensure sufficient irradiance, the light source was ≤ 3 cm from the sample surface (3). After chemiphotobleaching, cells were freeze-transferred to mirror-finished stainless steel microscope slides and subjected to Raman microspectroscopic analysis (Yakubovskaya *et al*, 2019)

Raman measurements, spectral analysis and imaging. Raman measurements were performed using a Renishaw® inVia™ confocal Raman microspectrophotometer configured with a modified upright Leica® DM2700™ fluorescence microscope and a computer-controlled motorized XYZ stage, a He-Ne laser for 633 nm excitation, and a thermo-electrically cooled 1024×256 CCD detector.

Spectra of individual virions and viral aggregates were acquired through a 100x dry objective lens (Leica 566202, HC PL Fluotar, 0.9 NA), with laser power about 4 mW at the sample, and 400 s total detector exposure time. Two-dimensional (2-D) mapping of label-free host cells was performed after chemiphotobleaching the entire sample for 2 h and an additional 1 to 3 min laser bleaching of each individual cells under 50x objective to remove weak residual autofluorescence. Raman chemical maps were acquired in StreamHR mode through our 100x dry objective lens. Laser excitation power at the sample was ~4 mW, x-y step size between spectra was 0.5 µm, and detector exposure time was 50 s at each point. Spectral data from each cell were processed as follows: All spectra were truncated to the region of interest (450 to 2000 cm^{-1}) and linear baseline correction was performed using WiRE™ 5.1 Renishaw® software. Further processing was performed with custom Python scripts as follows: all spectra were normalized by the highest phenylalanine (Phe) peak (1002 cm^{-1}) in an individual cell, truncated into three regions, 760-800 cm^{-1} (DNA peak), 980-1020 cm^{-1} (protein peak) , and the 1800-2000 cm^{-1} peak-free region used for analytical error determination. Within the corresponding region, peaks emanating from DNA (782 cm^{-1}) and protein (Phe peak, 1002 cm^{-1}) were fit with Voigt probability distribution profiles and linear baseline correction. Analytical uncertainty of peak intensities was estimated from the standard deviation of the noise observed in the peak-free region after linear baseline correction. During peak fitting, positions of the peaks were allowed to vary ± 2 cm^{-1} around 782 cm^{-1} (DNA) and 1002 cm^{-1} (protein).

To determine ^{13}C isotopic enrichment in SIP experiments, spectra from 8 ^{13}C-labeled *E. huxleyi* cells or 10 viral aggregates were averaged and then truncated to the 450 – 2000 cm^{-1} using the WiRE™ 5.1 Renishaw® software. Further processing was performed with custom Python scripts. Spectra were normalized to the highest peak which was set to a value of 1, then truncated over two intervals; the Phe peak region (950 - 1050 cm^{-1}) and the peak-free region (1800-2000 cm^{-1}) used to define the statistical uncertainty of the signal (noise level). Linear base-line correction was performed on the 1800-2000 cm^{-1} interval, standard deviation was computed and used in Python LMFIT peak-fitting procedure for error estimations. After baseline correction, the 950 - 1050 cm^{-1} interval was deconvolved by fitting region with five Voigt probability distribution profiles: 4 profiles corresponding to predicted isotopologues of the ^{13}C-labeled phenyl ring (0, 2, 4, and 6 six ^{13}C atoms

in the ring at 1002, 988, 977, and 966 cm^{-1}, respectively (Li *et al*, 2013)), and a peak at 1008 cm^{-1} that overlapped with the 1002 cm^{-1} peak using the Python LMFIT library. During the Non-Linear Least-Squares peak-fitting routine, peak positions of all four isotopologues were allowed to vary ± 1 cm^{-1}, and all peaks widths were allowed to vary simultaneously with possible limits from 0.1 to 10 cm^{-1}. Position of the 1008 cm^{-1} peak was limited between 1006 and 1015 cm^{-1} and the peak width allowed to vary from 1 to 6 cm^{-1}. The iterative fitting procedure ceased when the reduced Chi-square reached a minimum (successful fit) or after 5000 iterations without attaining the prescribed reduced Chi-square tolerance level ($1.0e^{-7}$) (unsuccessful fit). After successful fit completion, standard errors for fitted variables were automatically calculated. Fractional ^{13}C abundance of target (f_{target}) can be approximated as the intensity ratio of the sum of fitted peaks of the three enriched isotopologues, weighted by the number of ^{13}C atoms per isotopologue, divided by the sum of all intensities (Eqn. 1).

$$f_{target} = \frac{I_{966} + 0.67 \cdot I_{977} + 0.33 I_{988}}{I_{966} + I_{977} + I_{988} + I_{1002}} \qquad \text{Eqn. 1}$$

References

Bocklitz T, Kämmer E, Stöckel S, Cialla-May D, Weber K, Zell R, Deckert V, Popp J. (2014). Single virus detection by means of atomic force microscopy in combination with advanced image analysis. J Struct Biol. 188:30-8

Guillard R.R.L., and Ryther J.H. (1962) Studies of marine planktonic diatoms. I. *Cyclotella nana* Hustedt and Detonula confervacea Cleve. Canadian Journal of Microbiology 8, 229-239.

Li M., Huang W.E., Gibson C.M., Fowler P.W., and Jousset A. (2013) Stable Isotope Probing and Raman Spectroscopy for Monitoring Carbon Flow in a Food Chain and Revealing Metabolic Pathway. Analytical Chemistry 5, 1642

Taylor G.T., Li Z.Q., Suter E., Chow S. (2017). Modified filter-transfer-freeze (“FTF”) technique for Raman microspectroscopic analysis of single cells. Protocols.io, https://doi.org/10.17504/protocols.io.ikqccvw

Yakubovskaya E., Zaliznyak T., Martínez Martínez J., Taylor G.T. (2019) Virus purification protocol for Ehv-163 and other viruses. Protocols.io, dx.doi.org/10.17504/protocols.io.bcpvivn6)

Yakubovskaya E., Zaliznyak T., Martínez Martínez J., Taylor G.T. (2019). New chemiphotobleaching protocol for Raman spectroscopy. Protocols.io, dx.doi.org/10.17504/protocols.io.bchqit5w

2 Supplementary Figures and Tables

2.1 Supplementary Figures

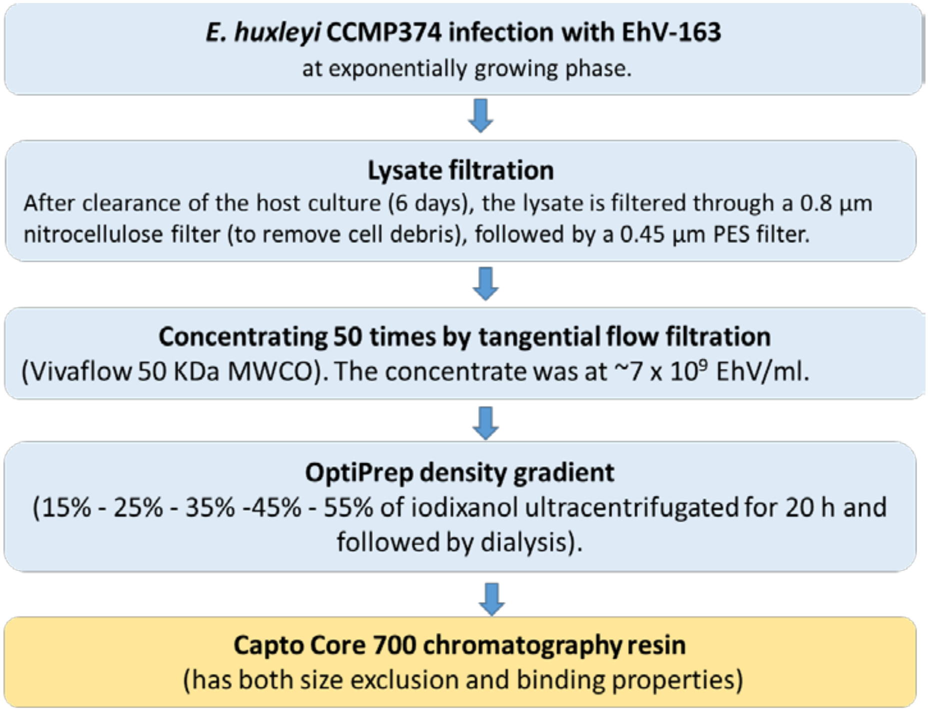

Supplementary Figure 1. EhV-163 purification protocol.

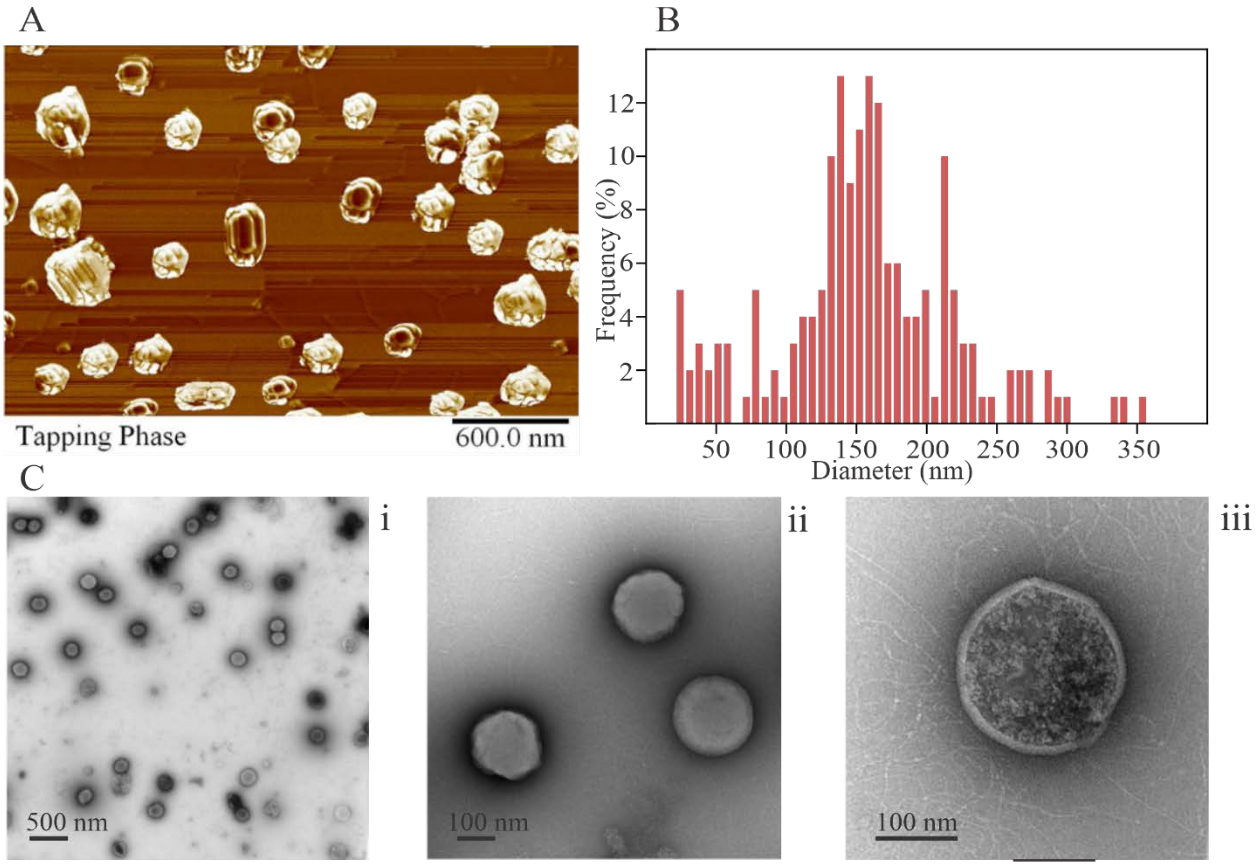

Supplementary Figure 2. (A) Atomic Force Microscopic (AFM) image of the purified enveloped EhV-163 virus sample (~1 x 10^{8} particles ml^{-1}) deposited on the surface of freshly cleaved high-grade mica, dried for 1 min at room temperature and washed twice with deionized water. Images were acquired on a Bruker® Innova™ AFM operating in tapping mode. **(B)** Particle size frequency distribution across multiple AFM fields of view (N = 174 particles). **(C)** TEM images of negatively stained EhV-163 viruses at 23,000x, 49,000x and 80,000x magnifications. EhV-163 particles were adsorbed to a glow discharged 400 mesh carbon-coated copper grid and stained with 1% (w/v) uranyl formate. Images were collected using a FEI BioTwinG2 TEM at HV 80kV.

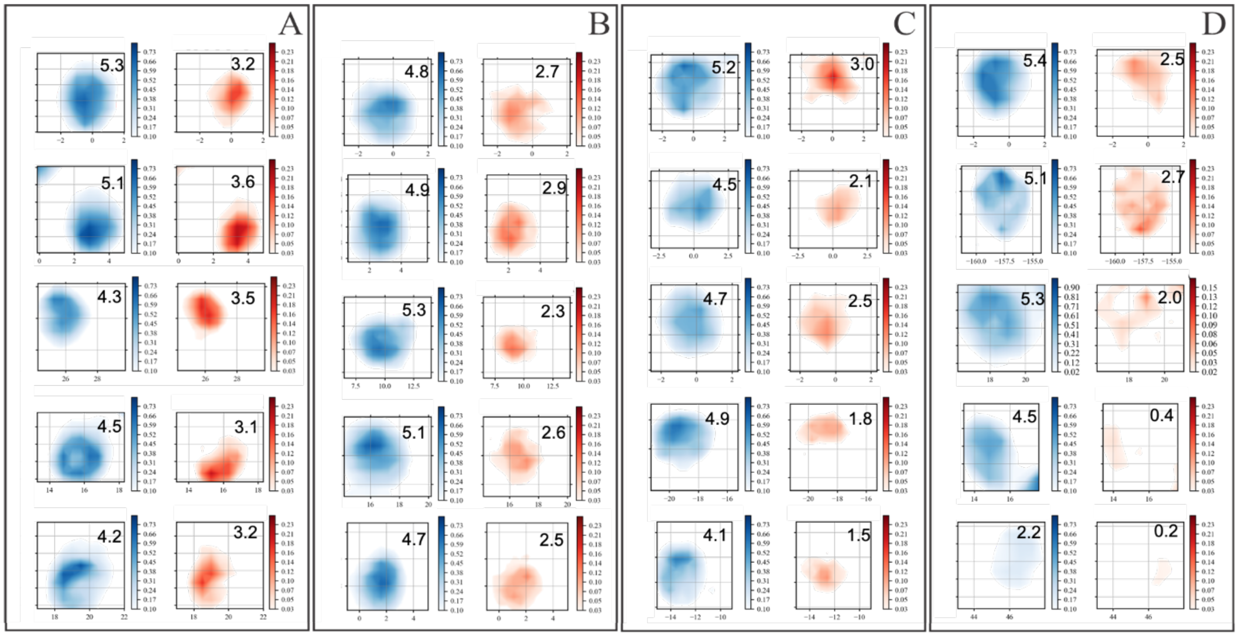

Supplementary Figure 3. Raman chemical maps of uninfected and infected *E. huxleyi* cells. Examples of two-dimensional Raman chemical maps of uninfected *E. huxleyi* cells **(A)**, and cells at 24 **(B)**, 48 **(C)**, and 72 **(D)** hours after infection with EhV-163. Intensities at 1002 cm^{-1} (protein - blue) and 782 cm^{-1} (DNA - red) were plotted using a standard shared color intensity scale for all cells. Integrated pixel intensities (arbitrary units) of each map are shown in the top right corner of each box.

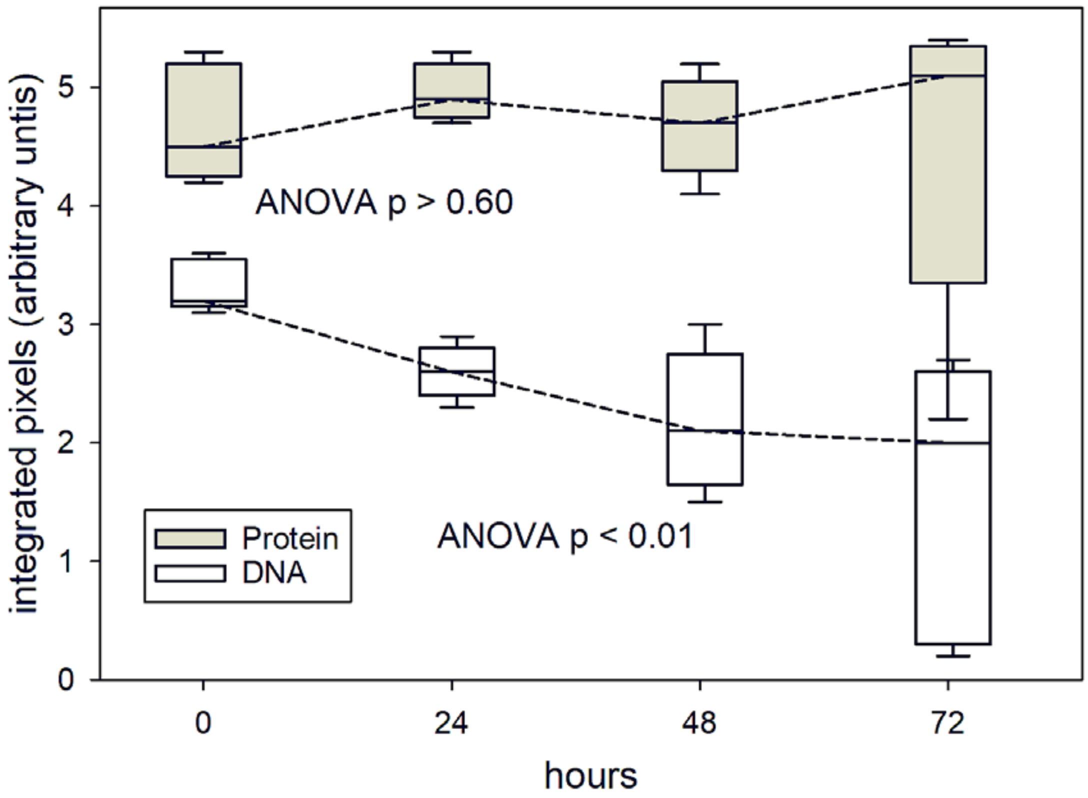

Supplementary Figure 4. Time course of integrated pixel intensities observed in protein and DNA portraits of *E. huxleyi* cells during EhV-163 infection development (summarizes data from Fig. SI3). Boxes represent the interquartile ranges of all five observations (25th - 75th percentiles). Internal horizontal lines and whiskers are medians and 10th to 90th percentiles, respectively. ANOVA demonstrates statistically significant decrease in overall intensity of DNA emission at 782 cm^{-1} ($p < 0.01$), whereas little systematic change is observed in phenylalanine emission at 1002 cm^{-1} observed ($p > 0.6$).
